# HM71224, a novel Bruton’s tyrosine kinase inhibitor, suppresses B cell and monocyte activation and ameliorates arthritis in a mouse model: a potential drug for rheumatoid arthritis

**DOI:** 10.1186/s13075-016-0988-z

**Published:** 2016-04-18

**Authors:** Jin Kyun Park, Joo-Yun Byun, Ji Ah Park, Yu-Yon Kim, Ye Ji Lee, Jeong In Oh, Sun Young Jang, Young Hoon Kim, Yeong Wook Song, Jeewoong Son, Kwee Hyun Suh, Young-Mi Lee, Eun Bong Lee

**Affiliations:** Division of Rheumatology, Department of Internal Medicine, Seoul National University College of Medicine, 101 Daehank-no Chongno-gu, Seoul, 03080 Korea; Hanmi Research Center, Hanmi Pharm.Co., Ltd., 550 Dongtangiheung-ro, Hwaseong-si, Gyeonggi-do 18469 Republic of Korea; Hanmi Pharm.Co., Ltd., 45 Bangi-dong, Songpa, Seoul, Gyeonggi-do 138-724 Republic of Korea

**Keywords:** B cells, Btk inhibitor, Inflammation, HM71224, Monocytes, Rheumatoid arthritis, Osteoclast

## Abstract

**Background:**

Bruton’s tyrosine kinase (Btk) is critical for activation of B cells and myeloid cells. This study aimed to characterize the effects of HM71224, a novel Btk inhibitor, both *in vitro* and in a mouse model of experimental arthritis.

**Methods:**

The kinase inhibition profile of HM71224 was analyzed. The in vitro effects of HM71224 on B cells and monocytes were analyzed by examining phosphorylation of Btk and its downstream signaling molecules, along with cytokine production and osteoclast formation. The in vivo effects of HM71224 were investigated in a mouse model of collagen-induced arthritis (CIA).

**Results:**

HM71224 irreversibly bound to and inhibited Btk (IC_50_ = 1.95 nM). The compound also inhibited the phosphorylation of Btk and its downstream molecules such as PLCγ2, in activated Ramos B lymphoma cells and primary human B cells in a dose-dependent manner. Furthermore, HM71224 effectively inhibited the production of tumor necrosis factor (TNF)-α, interleukin (IL)-6, and IL-1β by human monocytes, and osteoclast formation by human monocytes. Finally, HM71224 improved experimental arthritis and prevented joint destruction in a murine model of CIA.

**Conclusions:**

HM71224 inhibits Btk in B cells and monocytes and ameliorates experimental arthritis in a mouse model. Thus, HM71224 is a potential novel therapeutic agent for rheumatoid arthritis in humans.

**Electronic supplementary material:**

The online version of this article (doi:10.1186/s13075-016-0988-z) contains supplementary material, which is available to authorized users.

## Background

Rheumatoid arthritis (RA) is an autoimmune disease characterized by destructive joint inflammation and autoantibody production. A breach in self-tolerance of T and B lymphocytes leads to chronic activation of both innate and adaptive immune cells, with subsequent production of autoantibodies [1]. In addition, immune complexes (ICs) bind to macrophages to further drive production of inflammatory cytokines, which contribute to both local and systemic inflammation [2, 3]. Autoantibody-producing B cells are critical for the pathogenesis of RA; therefore, depleting B cells with the anti-CD20 antibody, rituximab, is an effective RA treatment [4]. However, depleting the entire B cell pool might have unforeseen long-term consequences as both immune-protective and regulatory cells that reside within the B cell pool will be lost [5]. Therefore, blockade of B cell receptor (BCR) signaling in more active (autoimmune) B cells might be a safer alternative. In this regard, selective inhibition of B cell activation might be of particular utility for the treatment of RA.

Bruton’s tyrosine kinase (Btk) is involved in BCR signaling during B cell activation [6]. Following BCR engagement, Src family kinases activate Btk, which then phosphorylates phospholipase-Cγ2 (PLCγ2), leading to calcium mobilization and activation of the nuclear factor-κB (NF-κB) and mitogen-activated protein (MAP) kinase pathways [7]. Activated B cells upregulate their cell surface expression of CD40, CD69, and CD86, all of which function in intercellular communication [8]. Interestingly, Btk (a member of the Tec kinase family) is involved in IC-mediated activation of monocytes and macrophages via Fcγ receptor (FcγR) binding. After binding to ICs, FcγRs activate Src kinases via the intracytoplasmic immunoreceptor tyrosine-based activation motif [9, 10]. In addition, Btk is involved in monocyte-derived osteoclast formation [11, 12].

HM71224 is a novel small molecule that covalently binds to Btk. The molecule was first identified from a large number of proprietary Btk inhibitors and chosen based on its potency, selectivity, and pharmacokinetic profile. The aims of this study were to examine the in vivo and in vitro effects of HM71224 on immune cell function and assess its suitability as a potential candidate for the treatment of RA.

## Methods

### Btk enzymatic activity assay

The indicated human kinases were examined in a kinase assay that measured their *Km* values for adenosine triphosphate. The assay was performed by a contract research organization (ThermoFisher Scientific, Waltham, MA, USA) using FRET-based Z’-Lyte (ThermoFisher Scientific) and tyrosine peptide substrates, according to the manufacturer’s instructions.

### Cell preparation

Ramos cells, the human Burkitt’s lymphoma cell line, was purchased from the American Type Culture Collection (Manassas, VA, USA). Primary human B cells were purified from healthy donors using either a RosetteSep Human B Cell Enrichment Cocktail (Stem Cell Technologies, Vancouver, BC, Canada) or a B cell isolation kit (Miltenyi Biotec, Bergisch Gladbach, Germany).

Blood was obtained from healthy donors after obtaining informed consent according to the Declaration of Helsinki. This was approved by the Institutional Review Board at Seoul National University Hospital. Human monocytes and plasmacytoid dendritic cells (PDCs) were isolated using human monocyte isolation kit II (Miltenyi Biotec) or human plasmacytoid dendritic isolation kit (Miltenyi Biotec). Murine bone marrow-derived macrophages (BMMs) were generated by culturing murine bone marrow cells in the presence of 10 ng/mL M-CSF (Sigma-Aldrich, St Louis, MO, USA) for 7 days.

### Analysis of Btk occupancy

After treatment with HM71224, cells were lysed and then incubated for 1 h with 1 μM of biotinylated probe (HM71224 derivative). The lysed cells were loaded into streptavidin-coated wells and then incubated with an anti-Btk antibody (1:1000; clone number D3H5, Cell signaling Technology, Danvers, MA, USA) for 1 h, followed by a secondary HRP-conjugated antibody (1:10000; EMD Millipore Corporation, Billerica, MA, USA) at room temperature for 1 h. After washing, 100 μL of colorimetric solution (R&D systems, Minneapolis, MN, USA) was added, and the reaction was stopped 30 minutes later by addition of 100 μL of sulfuric acid (0.2 moles/L). The plate was read with the absorbance reader at 450 nm.

To measure Btk occupancy by immunoprecipitation (IP), biotinylated HM71224 probes were preincubated with cell lysates for 1 h at 4 °C. Streptavidin-coated beads were then added, and the mixture was incubated overnight at 4 °C. Immunoblotting was then performed, and band density was measured using MultiGauge V3.0 software (FUJI FILM, Tokyo, Japan). Untreated biotinylated probes were set at 100 %.

### Immunoblotting

Ramos cells or human B cells were pretreated with the indicated concentrations of HM71224 and then stimulated with anti-IgM F(ab’)_2_ (Southern Biotech, Birmingham, AL, USA) for 10 minutes on ice. Cells were then lysed in RIPA buffer (Sigma-Aldrich), and the proteins were separated by SDS-PAGE (Bio-Rad Laboratories, Hercules, CA, USA), transferred onto polyvinylidene fluoride (PVDF) membranes, and immunoblotted with anti-pBtk Y223 (catalog number 5082; Cell Signaling Technology, Danvers, MA, USA), anti-pPLCγ2 Y759 (catalog number 3874; Cell Signaling Technology), anti-pPLCγ2 Y1217 (Cell Signaling Technology), anti-pERK T202/204 (197G2; Cell Signaling Technology), anti-Btk (C82B8; Cell Signal Technology), anti-PLCγ2 (catalog number 3872; Cell Signal Technology), anti-ERK1/2 (3A7; Cell Signaling Technology), and anti-GAPDH (FL-335; Santa Cruz Biotechnology, Santa Cruz, TX, USA) antibodies.

### Cytokine measurements

Human monocytes were stimulated with heat-inactivated ICs at 37 °C for 30 minutes in the presence of increasing concentrations of HM71224. Human PDCs were stimulated with lipopolysaccharide (LPS) or CpG ODN 2006 (ThermoFisher Scientific) for 48 h in the absence or presence of HM71224 (1 μM). The production of tumor necrosis factor (TNF)-α, interleukin (IL)-1β, and IL-6 was then measured using a commercial ELISA (R&D Systems).

### Flow cytometry analysis of cell activation and intracellular phospho-specific flow cytometry (phospho-flow)

Human PBMCs (10^6^ cells/mL) were allowed to rest at 37 °C for 2 h. The cells were then pretreated with 10-fold serial dilutions of HM71224 from 1000 nM to 1 nM for 2 h, followed by stimulation with anti-IgM F(ab’)_2_ for 24 h. To examine changes in the expression of surface activation markers, cells were stained with anti-CD3-FITC (SK7; BD Biosciences), anti-CD19-PE (HIB19; BD Biosciences), anti-CD40-PE-Cy7 (5C3; BD Biosciences), anti-CD69-V450 (FN50; BD Biosciences), and anti-CD86-PerCP-Cy5.5 (2331 (FUN-1); BD Biosciences) antibodies.

For phospho-flow, B cells were activated by crosslinking their BCRs with anti-human IgM antibodies (10 μg/mL; BD Biosciences) for 10 minutes at 37 °C. Cells were then fixed with 2 % paraformaldehyde at 37 °C for 12 minutes, permeabilized, and stained with anti-CD3-FITC (SK7; BD Biosciences), anti-CD20-PerCP-Cy5.5 (H1; BD Biosciences), anti-pBtk (Y223)-Alexa647 (N35-86; BD Biosciences), anti-pPLCγ2 (Y759)-Alexa647 (K86-689.37; BD Biosciences), anti-pSTAT1 (Y701)-Alexa647 (4a; BD Biosciences), anti-pSTAT3 (Y705)-Alexa647 (4/P-STAT3; BD Biosciences), anti-pSTAT5 (Y694)-Alexa647 (47; BD Biosciences), and anti-pERK1/2 (T202/204)-Alexa647 (20A; BD Biosciences) antibodies. The cells were then washed and analyzed on an LSR II flow cytometer (BD Biosciences). Data were analyzed using FlowJo software, version 8.8 (Treestar, Ashland, OR, USA).

### Osteoclast generation

Isolated human CD14 cells (1 × 10^5^ cells per well) were cultured in a 96-well plate at 37 °C in alpha-minimum essential medium (MEM) supplemented with 10 % FBS and 25 ng/mL M-CSF (Sigma-Aldrich) in the presence of increasing concentrations of HM71224. RANKL (100 ng/mL, Sigma-Aldrich) was added after 3 days. The medium was replaced every 3 days. On day 9, cells were fixed with 3 % formaldehyde and stained for tartrate-resistant acid phosphatase (TRAP) expression (Sigma-Aldrich). To drive osteoclast formation from bone morrow cells, BMMs (4 × 10^4^ per well) were stimulated with 30 ng/mL M-CSF and 100 ng/mL RANKL in the presence of increasing concentrations of HM71224 for 4 days. TRAP+ cells with three or more nuclei were counted as osteoclasts.

### Collagen-induced arthritis (CIA) model

All animal experimental protocols were approved by the animal care and use committee of the Hanmi Research Center, and performed in accordance with approved guidelines. Male DBA/1 J mice (9 weeks old; n = 7 per group) were immunized with an emulsion of Complete Freund’s Adjuvant and bovine type II collagen (CII) (total volume, 0.07 mL) via intradermal injection at the base of the tail (day 0). Three weeks later, the mice received a booster immunization comprising bovine CII emulsified in Incomplete Freund’s Adjuvant. Mice received either oral HM71224 (0, 1, 3, 10, or 30 mg/kg) or dexamethasone (0.2 mg/kg) once a day for 2 weeks, starting on day 10 after the booster immunization. The arthritis score and body weight were measured three times per week for 2 weeks after administration of the drugs (Additional file 1: Figure S1). The arthritis score was determined by grading each paw from 0 to 4 based on erythema, swelling, and flexion of the joint and was expressed as the sum of the scores of all four paws [13]. Serum levels of IL-6 were measured by Luminex analysis in a bead-based immunoassay (EMD Millipore Corporation). Serum levels of anti-CII and total IgG were measured by ELISA (Chondrex, Seattle, WA, USA; Bethyl Laboratories, Montgomery, TX, USA).

### X-ray micro-computed tomography analysis (micro-CT)

Micro-CT of the hind limbs was performed using a Siemens Inveon Micro-CT/PET scanner (Siemens Medical Solutions, Knoxville, TN, USA).

### Histopathologic assessment

The hind legs of each mouse were fixed with 10 % formalin, decalcified in 5 % formic acid, and embedded in paraffin. Sections were then cut and stained with hematoxylin and eosin (H&E). The bone erosion index was scored from 0 to 4 according to the following criteria: 0 = no erosion, 1 = mild (focal subchondral erosion), 2 = moderate (multiple subchondral erosions), 3 = high (as above and focal erosion of talus), and 4 = maximum (multiple erosions of tarsal and metatarsal bones).

### Statistical analysis

Data were expressed as the mean ± SEM. Statistical analyses were performed using one-way analysis of variance (ANOVA), followed by Dunnett’s multiple comparison tests or the Kruskal-Wallis test as appropriate (when data did not pass the Shapiro-Wilk test for normality). All statistical analyses were performed using Prism version 6.0 (GraphPad, La Jolla, CA, USA).

## Results

### HM71224 is a potent and selective inhibitor of Btk

**Fig. 1:**
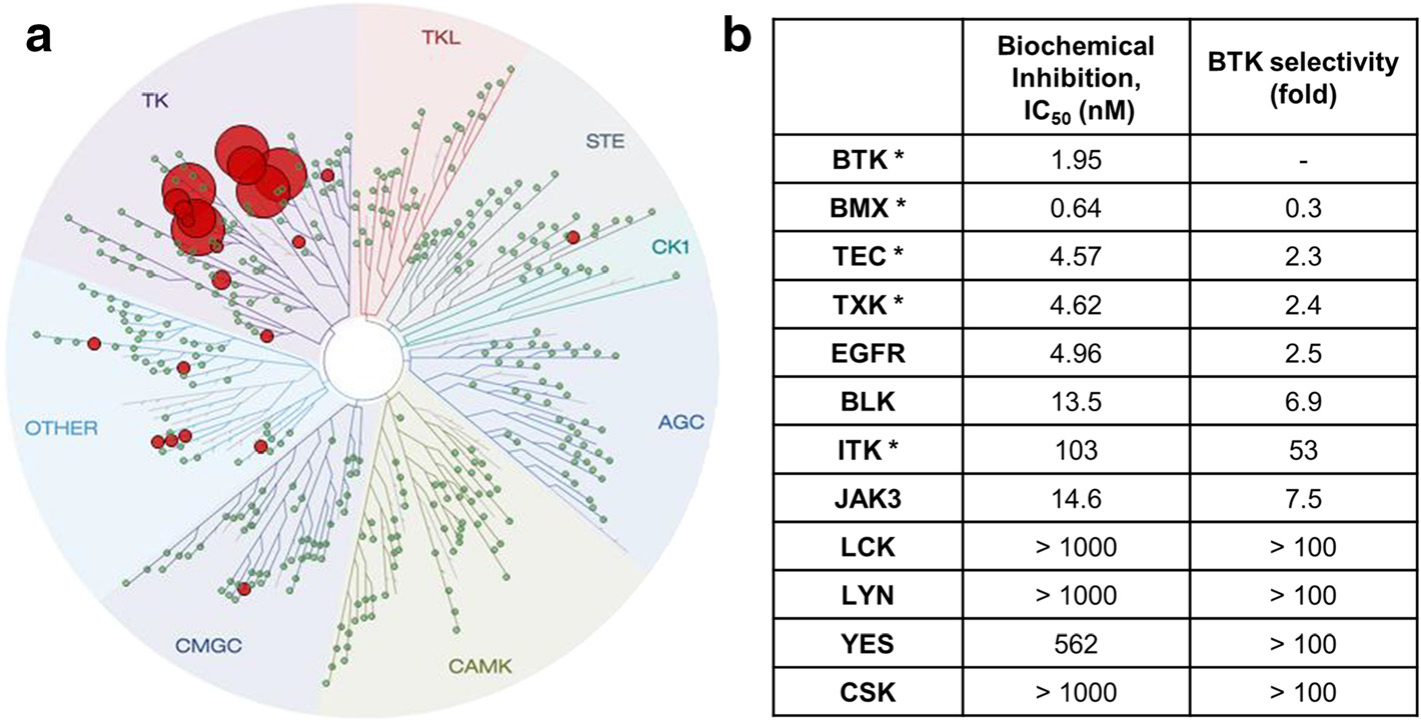
Kinase selectivity of the Bruton’s tyrosine kinase (Btk) inhibitor, HM71224. **a** Kinome Binding Tree Spot analysis of HM71224 based on Scan Max data (DiscoveRx). HM71224 selectively bound to Btk (a TEC family kinase) at a concentration of 2 μM (indicated by red circles). **b** Selectivity of Btk for a screened panel of kinases. *TEC family kinase (TFK), *EGFR* epidermal growth factor receptor, *JAK* Janus kinase, *BMX* bone marrow kinase on chromsome X, *TK* tyrosine kinase, *TKL* tyrosine kinase-like, *STE* homolog of Sterile, *CK1* Casein kinase 1, *AGC* protein kinase A, G, and C families, *CAMK* Ca2+/calmodulin-dependent protein kinase, CMGC cyclin-dependent kinases (CDKs), mitogen-activated protein kinases (MAP kinases), glycogen synthase kinases (GSK), CDK-like kinases, *BLK* B-cell lymphocyte kinase, *ITK* IL2-Inducible T-Cell Kinase, *JAK3* Janus kinase 3, *LCK* lymphocyte-specific protein tyrosine kinase, *CSK* C-Src Tyrosine Kinase

To determine the biochemical selectivity of HM71224, we tested more than 85 kinases using the fluorescence resonance energy transfer (FRET) method. As summarized in Fig. 1, HM71224 was highly selective for Btk: HM71224 inhibited Btk with an IC_50_ of 1.95 nM. HM71224 inhibited other kinases, including BMX, TEC, and TXK, which carry a conserved cysteine in the binding pocket (Additional file 1: Table S1). HM71224 had some inhibitory effect on the epidermal growth factor receptor (EGFR) in biochemical sense (Fig. 1b). However, more than 1000 nM of HM71224 was required to inhibit 50 % of cellular growth in EGFR-overexpressed A431 cells (Additional file 1: Figure S2A) or phosphorylation of EGFR (Additional file 1: Figure S2B).

### HM71224 is a potent inhibitor of B cell signaling and activation

**Fig. 2:**
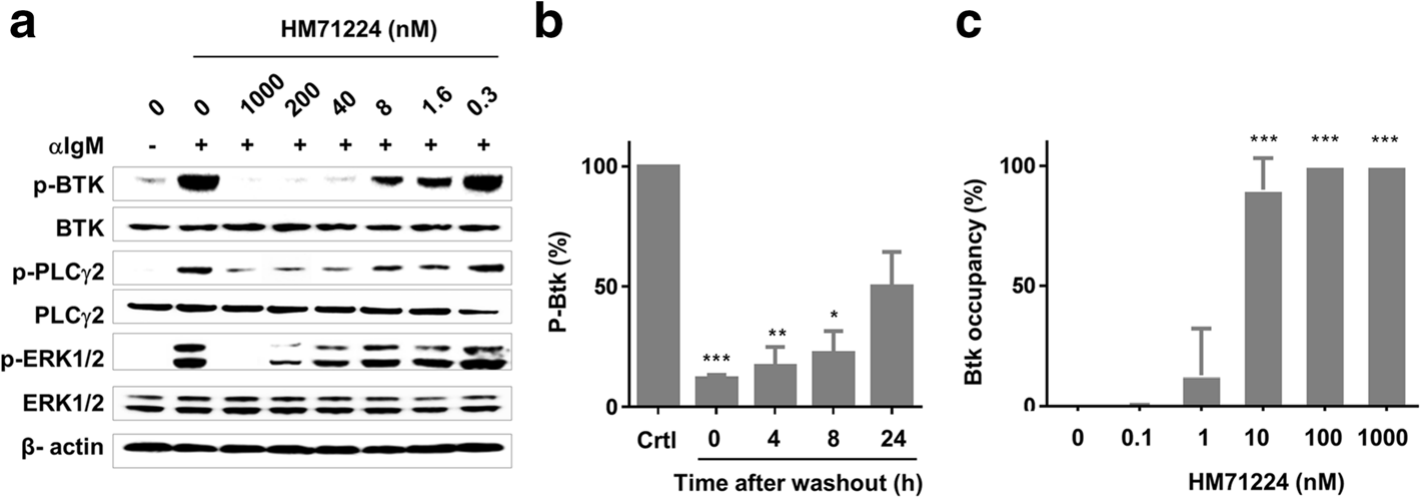
Effect of HM71224 on Bruton’s tyrosine kinase (*Btk*) signaling. **a** The inhibitory effects of HM71224 on B cell receptor (BCR) signaling in the Ramos cell line. Ramos cells were stimulated with anti-IgM F(ab’)_2_ fragments in the presence of the indicated doses of HM71224. **b** Time-dependent restoration of Btk phosphorylation in Ramos cells after HM71224 washout. **c** Btk occupancy by HM71224 on Ramos cells. *P < 0.05, **P < 0.01, and ***P < 0.001 vs. untreated control (*crtl*). *ERK* extracellular signal-regulated kinase

The Ramos B cell lymphoma cell line was chosen to study the inhibitory effects of HM71224 on the BCR signaling cascade as it endogenously expresses Btk [14]. Crosslinking of BCRs with anti-IgM F(ab’)_2_ induced phosphorylation of Btk and PLCγ2. However, phosphorylation of Btk and PLCγ2 decreased markedly in the presence of HM71224 (Fig. 2a). Next, we investigated whether HM71224 irreversibly inhibited Btk. Ramos cells were incubated with 100 nM HM71224 for 1 h. After washing, the cells were stimulated with anti-IgM F(ab’)_2_. The inhibitory effect of BTK phosphorylation by HM71224 treatment lasted at least 24 h (Fig. 2b). Biotinylated probe bound to free Btk in a dose-dependent manner; approximately 90 % of Btk molecules were occupied by HM71224 at 10 nM, and close to 100 % at 100 nM (Fig. 2c).

**Fig. 3:**
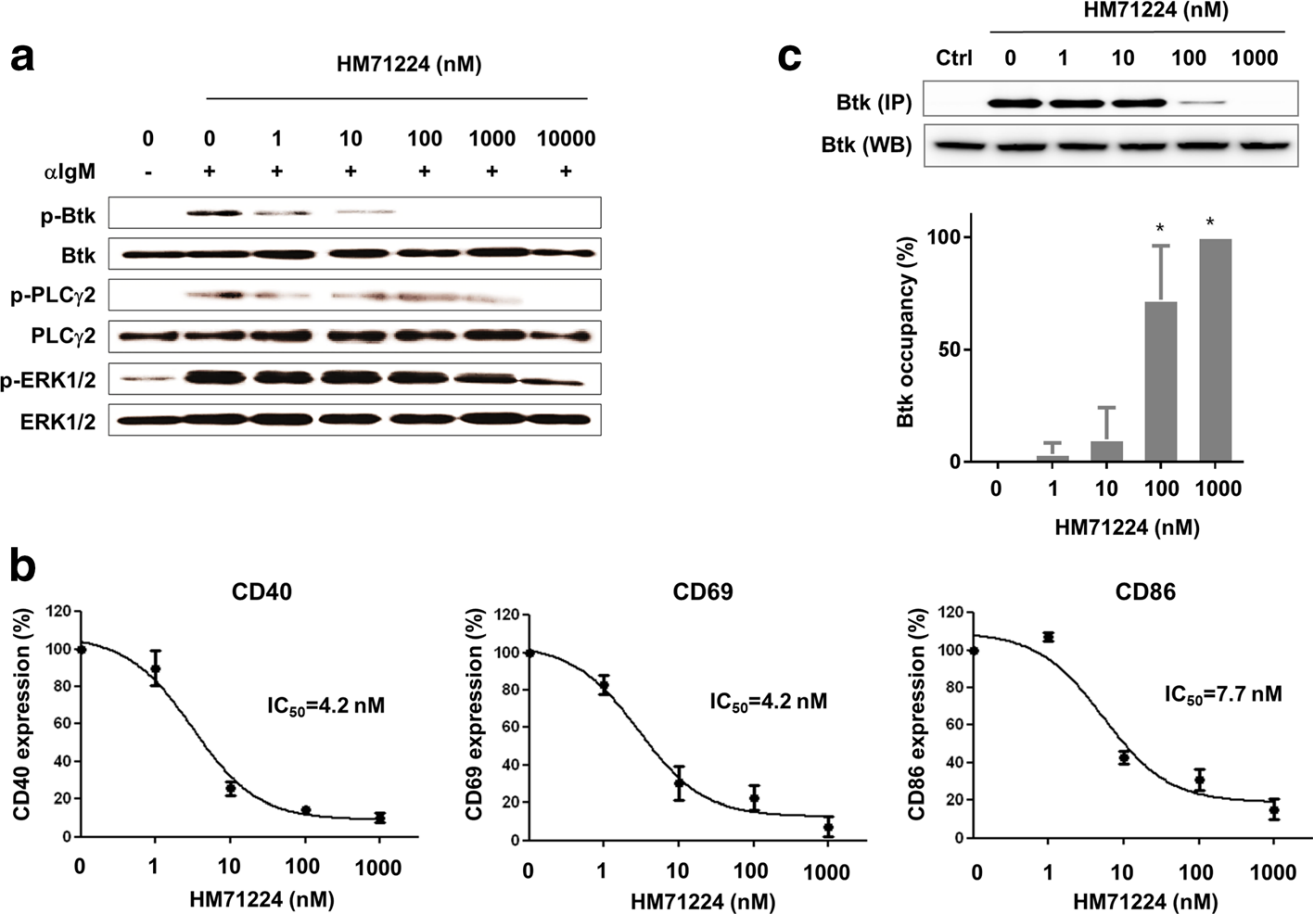
HM71224 inhibits human B cell activation. **a** Healthy primary human CD19+ B cells (n = 3) were activated with anti-IgM F(ab’)_2_ fragments in the presence of increasing concentrations of HM71224. Phosphorylation of Bruton’s tyrosine kinase (*Btk*) and PLCγ2 in human B cells was inhibited in a dose-dependent manner. The immunoblot shown is representative of three independent experiments. **b** HM71224 inhibits the surface expression of CD40, CD69, and CD86 by activated B cells. **c** Percentage of Btk sites on human peripheral blood mononuclear cells (PBMCs) occupied by HM71224, as measured by a biotinylated probe. *P < 0.001 vs*.* 0 nM HM71224. *ERK* extracellular signal-regulated kinase

We next examined the effects of HM71224 on primary human cells. Upon BCR ligation, phosphorylation of Btk was followed by rapid phosphorylation of PLCγ2. HM71224 effectively inhibited the phosphorylation of Btk and PLCγ2 (Fig. 3a). This inhibitory effect was confirmed by phospho-flow cytometry (Additional file 1: Figure S3). In addition, the surface expression of CD40, CD69, and CD86 by activated primary human B cells was markedly inhibited by HM71224 (IC_50_ = 4.2 nM for CD40; IC_50_ = 4.2 nM for CD69; and IC_50_ = 7.7 nM for CD86) (Fig. 3b). HM71224 occupancy of Btk in primary human cells was dose-dependent: 100 nM HM71224 occupied >70 % of Btk sites (Fig. 3c).

### Effect of HM71224 on Fc gamma receptor (FcγR) and toll-like receptor (TLR) signaling in primary human cells

**Fig. 4:**
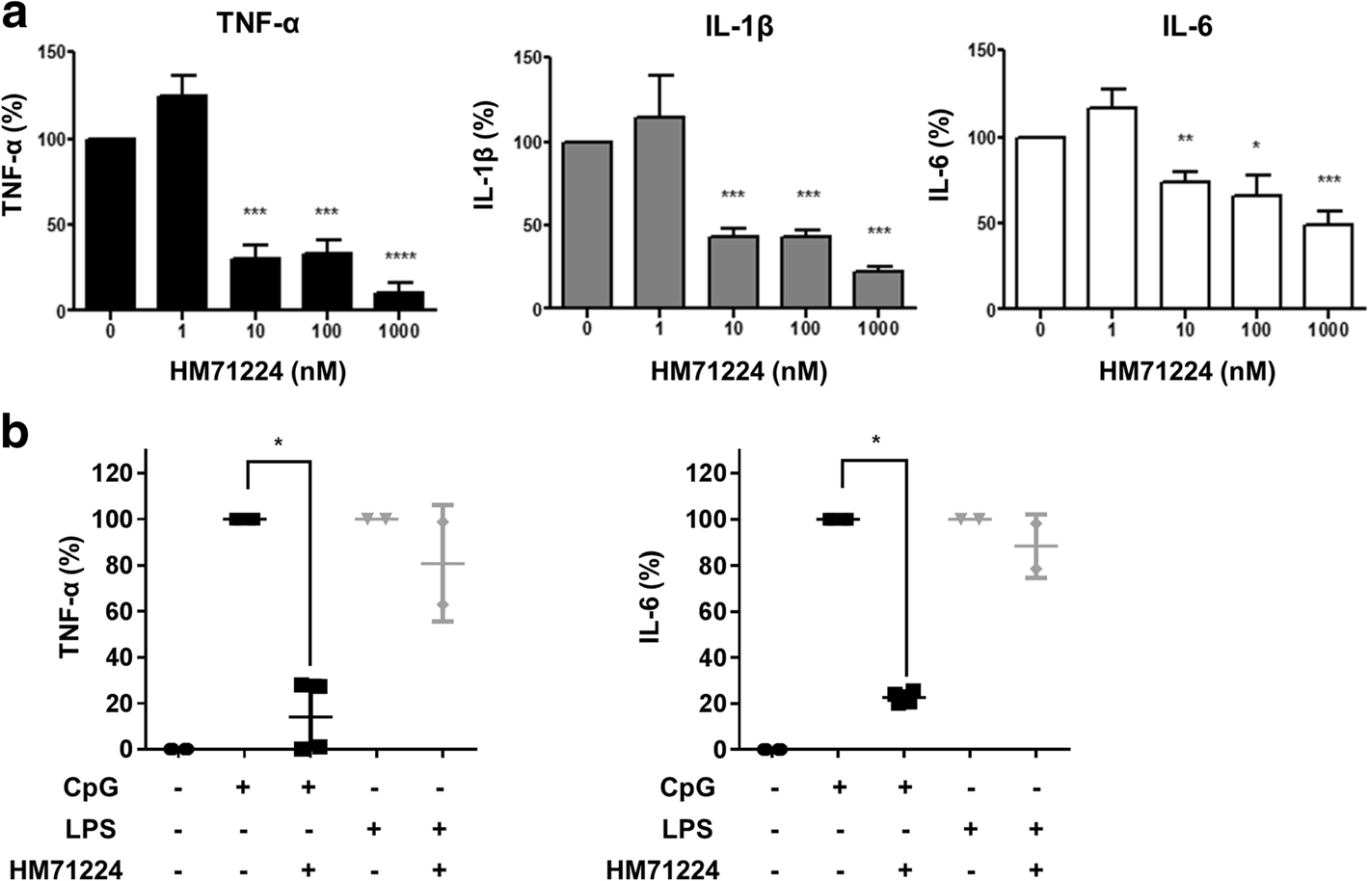
HM71224 inhibits cytokine production by human peripheral blood monocytes and plasmacytoid dendritic cells. **a** Human CD14+ monocytes were isolated from healthy donors (n = 3) and stimulated with heat-inactivated immune complexes (*ICs*) in the presence of HM71224. The levels of the indicated cytokines in the culture supernatant were then measured by ELISA at 5 h post stimulation. HM71224 significantly reduced the levels of secreted cytokines in a dose-dependent manner. **b** Human peripheral blood dendritic cells were isolated from healthy donors (n = 2) and stimulated with CpG oligodeoxynucleotides (CpG ODN) or lipopolysaccharide (LPS). The levels of the indicated cytokines in the culture supernatant were then measured by ELISA at 48 h post stimulation.*P < 0.05, **P < 0.01, and ***P < 0.001 vs. no treatment. *LPS* lipopolysaccharide

Btk is involved in signal transduction by FcγRs expressed by myeloid lineage cells such as monocytes, macrophages, and neutrophils [15]. Therefore, we next examined the effect of HM71224 on inflammatory cytokine production by human monocytes. Human CD14+ cells were isolated from healthy volunteers and stimulated with heat-inactivated ICs in the presence of increasing concentrations of HM71224. The production of TNF-α, IL-1β, and IL-6 was inhibited in a dose-dependent manner (Fig. 4a). Btk also plays a crucial role in activating human PDCs via the TLR9 signaling pathway [16]. As expected, stimulation of human PDCs with either LPS (a TLR4 agonist) or CpG oligodeoxynucleotides (CpG ODN, a TLR9 agonist) induced the production of TNF-α and IL-6. HM71224 inhibited CpG-mediated cytokine production but not that mediated by LPS (Fig. 4b).

### HM71224 inhibits osteoclast formation

**Fig. 5:**
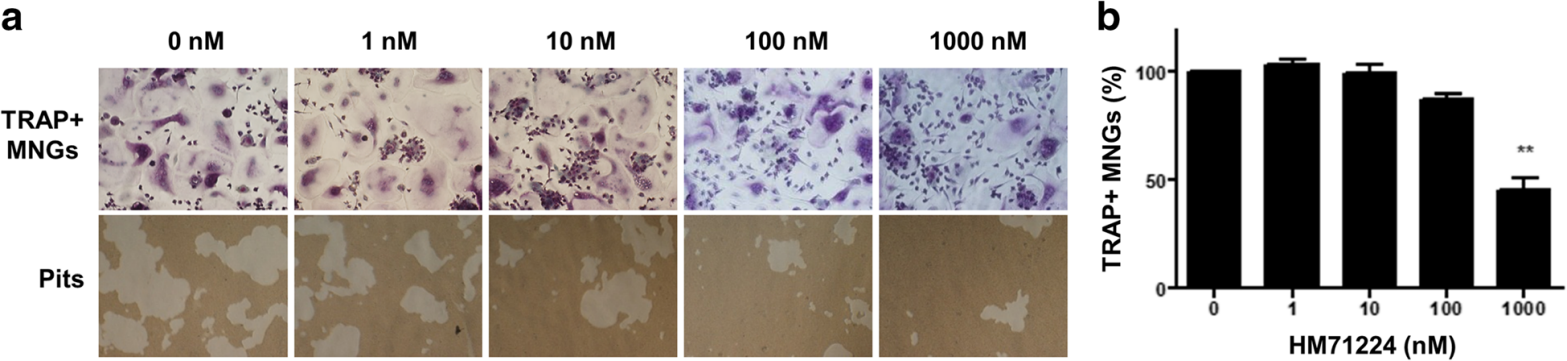
HM71224 inhibits osteoclastogenesis. **a** Representative images of osteoclastogenesis. Human CD14+ monocytes were cultured with the indicated concentrations of HM71224 during osteoclast differentiation (magnification × 100). **b** Dose-dependent inhibition of tartrate resistant alkaline phosphatase (*TRAP*)+ cells by HM71224. *P < 0.05 and **P < 0.01 vs. no treatment. MNGs is multinucleated giant cells

Osteoclasts were generated from human CD14+ cells. Numerous TRAP+ osteoclasts were generated in the absence of HM71224; these cells were able resorb the bone matrix and form pits. HM71224 inhibited osteoclast formation in a dose-dependent manner (Fig. 5). However, approximately 50 % of inhibition was reached at 1000 nM HM71224. Similarly, HM71224 inhibited osteoclast formation from murine BMMs (Additional file 1: Figure S4).

### Therapeutic efficacy of HM71224 in mice with experimental arthritis

**Fig. 6:**
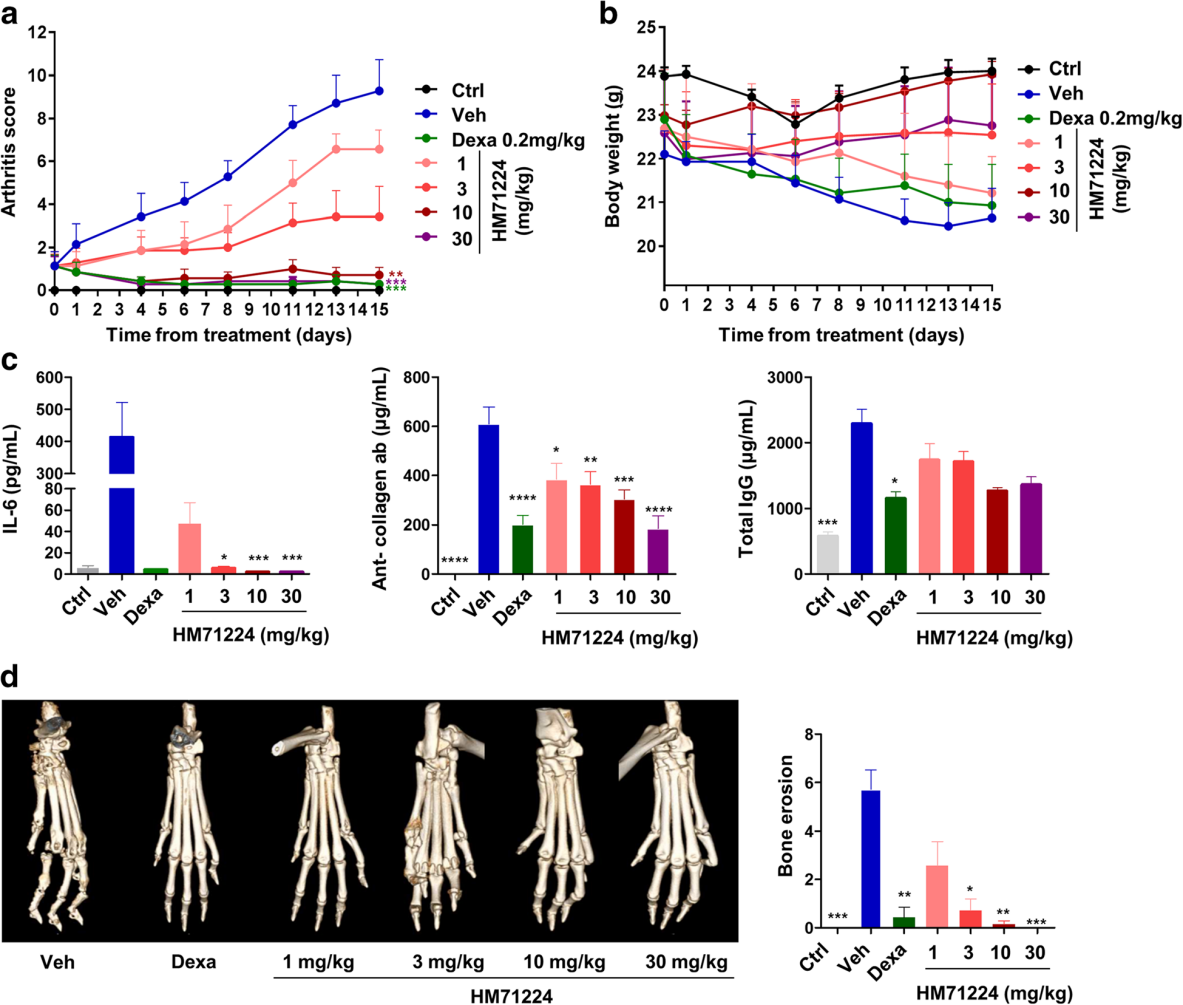
HM71224 ameliorates symptoms in a mouse model of collagen-induced arthritis (CIA). As described in “Methods”, arthritis was induced by immunization with collagen. From 10 days after the second immunization, mice were treated with oral HM71224, dexamethasone (*Dexa*), or vehicle (*Veh*). Compared with vehicle, treatment with HM71224 led to dose-dependent improvements in the clinical arthritis score (**a**) and body weight (**b**). **c** HM71224 reduced serum IL-6 (*left*), and anti-type II collagen (*middle*) and total IgG levels (*right*). **d** The bone erosion was assessed on a hematoxylin and eosin section. HM71224 treatment reduced the degree of bone erosion. *P < 0.05, **P < 0.01, and ***P < 0.001 vs. CIA mice treated with vehicle. *Ctrl* (healthy) control

Finally, we explored whether HM71224 could ameliorate the signs and symptoms of experimental arthritis. Mice treated with HM71224 had lower arthritis scores than untreated CIA mice (*P* < 0.05 at 10 mg/kg and *P* < 0.01 at 30 mg/kg). The efficacy of HM71224 at 10 mg/kg and 30 mg/kg was comparable with that of dexamethasone at 0.2 mg/kg (Fig. 6a). HM71224 at a dose of 3 mg/kg or higher prevented weight loss (Fig. 6b) (Additional file 1: Table S2). In addition, serum IL-6 levels and the levels of circulating anti-collagen antibodies were significantly lower in the HM71224 group than in the untreated group. The total IgG level tended to be lower after HM71224 treatment (Fig. 6c). HM71224 markedly reduced erosive bone changes and prevented bone loss (Fig. 6d).

## Discussion

The current study demonstrated that the novel Btk inhibitor HM71224 effectively suppressed activation of B cells and monocytes, as well as osteoclast formation, by directly blocking Btk and its downstream signaling cascades. Furthermore, HM71224 ameliorated the signs and symptoms of experimental arthritis and prevented cartilage and bone destruction in a murine model of arthritis.

Tyrosine kinases are important therapeutic targets for diseases such as cancer and autoimmunity [17–19]. Here, we showed that HM71224 effectively blocked the phosphorylation of Btk and its downstream molecule, PLCγ2, in a human B cell lymphoma cell line and in primary human B cells following BCR activation. This inhibition of BCR signaling translated into reduced expression of CD40, CD69, and CD86 on the B cell surface (Fig. 3). CD40 and CD69 are critical for B cell survival and proliferation, whereas CD86 functions as a costimulatory molecule for T cell activation. Furthermore, HM71224 inhibited FcγR-mediated activation of monocytes and effectively suppressed their production of inflammatory cytokines [10, 12]. Of note, HM71224 did not inhibit JAK/STAT signaling following T cell activation (Additional file 1: Figure S5). Taken together, these data show that HM71224 is a selective Btk inhibitor that can suppress both B cells and monocytes.

Because B cells are crucial for the development of RA [20, 21], the anti-CD20 antibody rituximab, which depletes CD20+ B cells, is an effective RA treatment [4, 22–24]. Here, we found that HM71224 effectively ameliorated arthritis and prevented joint destruction in a mouse model of CIA. This was accompanied by reduced production of anti-collagen II antibodies, suggesting that HM71224 suppresses autoimmune B cells. This finding is consistent with prior studies showing that other Btk inhibitors are effective against experimental arthritis [25, 26]. As HM71224 inhibits activation of B cells, monocytes, and PDCs, further studies are needed to examine whether the therapeutic efficacy of Btk inhibition exceeds that of B cell depletion alone. Of note, the inhibition of osteoclastogenesis required 100 times the IC_50_ reported to inhibit human monocytes (Figs. 4 and 5), suggesting that the inhibition of human osteoclastogenesis might represent an off-target effect of HM72114.

Dose-dependent inhibition of Btk by HM71224 might regulate the activation threshold of immune cells. As lymphoma cells are more dependent on Btk signaling, one might speculate that chronically activated B cells in individuals with autoimmune diseases are also more dependent on Btk [27]. In this case, autoimmune cells would be preferentially affected by Btk inhibition. On the other hand, as long-lived plasma cells express low levels of Btk, the production of protective antibodies would less likely be affected by Btk inhibition [28].

Because RA treatments are associated with an increased risk of infection, the benefits of adding immunosuppressive treatments must be carefully weighed against the infection risk [29, 30]. In this regard, it is important that HM71224 did not affect TLR4-mediated signaling (Fig. 4b), as TLR4 is a main receptor expressed by innate immune cells such as neutrophils and monocytes, both of which recognize and mount immune responses against gram-negative bacteria [31–33]. Therefore, HM71224 would be less likely to interfere with antibacterial defense. In addition, in cases of an active infection, HM71224 can be withheld to allow restoration of normal immune responses (Fig. 2b). Interestingly, HM71224 did block TLR9-mediated signaling. Of note, the TLR9 pathway is particularly important in systemic lupus erythematosus (SLE), where autoantibody production and cytokine production by PDCs are crucial [34]. In this respect, HM71224 would be an effective treatment for SLE as it also inhibits autoantibody production and cytokine production by PDCs. However, the potent inhibition of TLR9 might be also associated with an impaired antiviral immune response, which involves production of type I interferons following the recognition of viral nucleic acids by endoplasmic TLR7 and TLR9 [35]. Nevertheless, HM71224 might have therapeutic utility for other diseases such as anti-neutrophil cytoplasmic antibody-associated vasculitis, in which B cell depletion is effective [36–40].

## Conclusions

HM71224, a novel Btk inhibitor, suppresses the activation of B cells, myeloid cells, and dendritic cells. It also ameliorates experimental arthritis and prevents joint destruction in an animal model. Currently, a clinical trial is planned to investigate the efficacy and safety of HM71224 in patients with RA.

## Additional file

Additional file 1: Table S1. Sequence alignment of kinases. **Table S2.** Arthritis score and body weight during treatment period. **Figure S1.** Experimental design of collagen-induced arthritis. **Figure S2.** Effect of HM71224 on EGFR signaling. **Figure S3.** HM71224 inhibits Btk phosphorylation. **Figure S4.** HM71224 inhibits osteoclastogenesis. **Figure S5.** Effect of HM71224 on human T cell signaling. (DOCX 1545 kb)

## Abbreviations

BCR: B cell receptor
BMMs: murine bone marrow-derived macrophages
Btk: Bruton’s tyrosine kinase
CII: bovine type II collagen
CIA: collagen-induced arthritis
EGFR: epidermal growth factor receptor
ELISA: enzyme-linked immunosorbent assay
ERK: extracellular signal-regulated kinase
FcγR: Fcγ receptor
FRET: fluorescence resonance energy transfer
ICs: immune complexes
IL: interleukin
IP: immunoprecipitation
JAK: Janus kinase
LPS: lipopolysaccharide
MAP: mitogen-activated protein
micro-CT: micro-computed tomography
NF-κB: nuclear factor-κB
PDC: plasmacytoid dendritic cell
PLCγ2: phospholipase-Cγ2
RA: rheumatoid arthritis
SLE: systemic lupus erythematosus
TLR: toll-like receptor
TNF: tumor necrosis factor
TRAP: tartrate-resistant acid phosphatase
