# Supplementary material for: HM71224, a novel Bruton’s tyrosine kinase inhibitor, suppresses B cell and monocyte activation and ameliorates arthritis in a mouse model: a potential drug for rheumatoid arthritis
Source: Arthritis Res Ther. 2016 Apr 18;18:91. doi: 10.1186/s13075-016-0988-z (PMC4835877; doi:10.1186/s13075-016-0988-z)

**Additional file 1**

**Table S1. Sequence alignment of kinases.**

| **Kinase** | **Sequence alignment** |
| --- | --- |
| BTK | RPIFIITEYMANGCLLNYLREMRHR |
| BMX | YPLYLVTEYLSNGCLLNYIRSHGKG |
| TEC | KPIYIVTEFMERGCLLNFLRQRQGH |
| TXK | KPLYIVTEFMENGCLLNYLRENKGK |
| ITK | APICLVFEFMEHGCLSDYLRTGRGL |
| EGFR | STVQLITQLMPFGCLLDYVREHKDN |
| JAK3 | PELRLVMEYLPSGCLRDFLQRHRAR |
| BLK | EPIYIVTEYMARGCLLDFLKTDEGS |

**Table S2. Arthritis score and body weight during treatment period.**

|  |  | D0 | D1 | D4 | D6 | D8 | D12 | D13 | D15 |
| --- | --- | --- | --- | --- | --- | --- | --- | --- | --- |
| Ctrl | AS | 0.0±0.0 | 0.0±0.0 | 0.0±0.0 | 0.0±0.0 | 0.0±0.0 | 0.0±0.0 | 0.0±0.0 | 0.0±0.0 |
|  | Wt | 23.9±0.2 | 23.9±0.2 | 23.4±0.2 | 22.8±0.2 | 23.4±0.3 | 23.8±0.3 | 24.0±0.3 | 24.0±0.3 |
| Veh | AS | 1.1±0.7 | 2.1±1.0 | 3.4±1.1 | 4.1±0.9 | 5.3±0.7 | 7.7±0.9 | 8.7±1.3 | 9.3±1.4 |
|  | Wt | 22.1±0.5 | 21.9±0.5 | 21.9±0.6 | 21.4±0.6 | 21.1±0.5 | 20.6±0.5 | 20.5±0.5 | 20.6±0.7 |
| Dexa | AS | 1.1±0.5 | 0.9±0.5 | 0.4±0.2 | 0.3±0.2 | 0.3±0.2 | 0.3±0.2 | 0.4±0.3 | 0.3±0.2 |
|  | Wt | 22.9±0.4 | 22.1±0.4 | 21.6±0.3 | 21.5±0.3 | 21.2±0.3 | 21.4±0.3 | 21.0±0.3 | 20.9±0.4 |
| HM  (1mg/kg) | AS | 1.1±0.7 | 1.1±0.7 | 1.9±0.9 | 2.1±1.1 | 2.9±1.1 | 5.0±1.0 | 6.6±0.7 | 6.6±0.9 |
|  | Wt | 22.7±0.5 | 22.5±0.5 | 22.2±0.6 | 21.9±0.5 | 22.1±0.5 | 21.6±0.5 | 21.4±0.4 | 21.2±0.3 |
| HM  (3mg/kg) | AS | 1.1±0.3 | 1.3±0.7 | 1.9±0.6 | 1.9±0.6 | 2.0±0.7 | 3.1±0.9 | 3.4±1.2 | 3.4±1.4 |
|  | Wt | 22.7±0.4 | 22.3±0.5 | 22.2±0.4 | 22.4±0.4 | 22.5±0.4 | 22.6±0.4 | 22.6±0.4 | 22.5±0.4 |
| HM  (10mg/kg) | AS | 1.1±0.6 | 0.9±0.3 | 0.4±0.2 | 0.6±0.4 | 0.6±0.3 | 1.0±0.4 | 0.7±0.4 | 0.7±0.4 |
|  | Wt | 23.0±0.2 | 22.8±0.3 | 23.2±0.3 | 23.0±0.3 | 23.2±0.3 | 23.5±0.3 | 23.8±0.3 | 23.9±0.3 |
| HM  (30mg/kg) | AS | 1.1±0.5 | 0.9±0.3 | 0.3±0.2 | 0.3±0.2 | 0.4±0.2 | 0.4±0.2 | 0.4±0.3 | 0.3±0.2 |
|  | Wt | 22.6±0.5 | 22.0±0.5 | 22.1±0.4 | 22.1±0.4 | 22.4±0.4 | 22.5±0.4 | 22.9±0.5 | 22.8±0.4 |

Data are in mean and standard deviation. AS, arthritis score; Wt weight in (gram); Ctrl, control; D, day; Dexa, dexamethasone; HM, HM71224; Veh, vehicle.

**Figure S1. Experiment design of collagen induced arthritis.** Untreated DBA/1J mice (n=7) served as healthy controls (Ctrl). DBA/1J mice (n= 56) were immunized Day 0 and day 21. On day 10 after boosting (i.e. day 31), the induction of CIA was evaluated. Mice with arthritis score 4 (n= 8) or without a weight loss (n= 4) and mice that died during the immunization (n=2) were excluded. Immunized mice (n=42) were divided into 6 groups (n=7 in each group). Each group was treated with vehicle, dexamethasone (0.2 mg/kg), and HM71224 (1, 3, 10 and 30 mg/kg), respectively, for 14 days. The arthritis score and weight of the mice were measured on days 0, 1, 4, 6, 8, 11, 13 and 15 after the active treatment begin. CIA, collagen induced arthritis. Crtl, control; Veh, Dexa; dexamethasone vehicle.

**
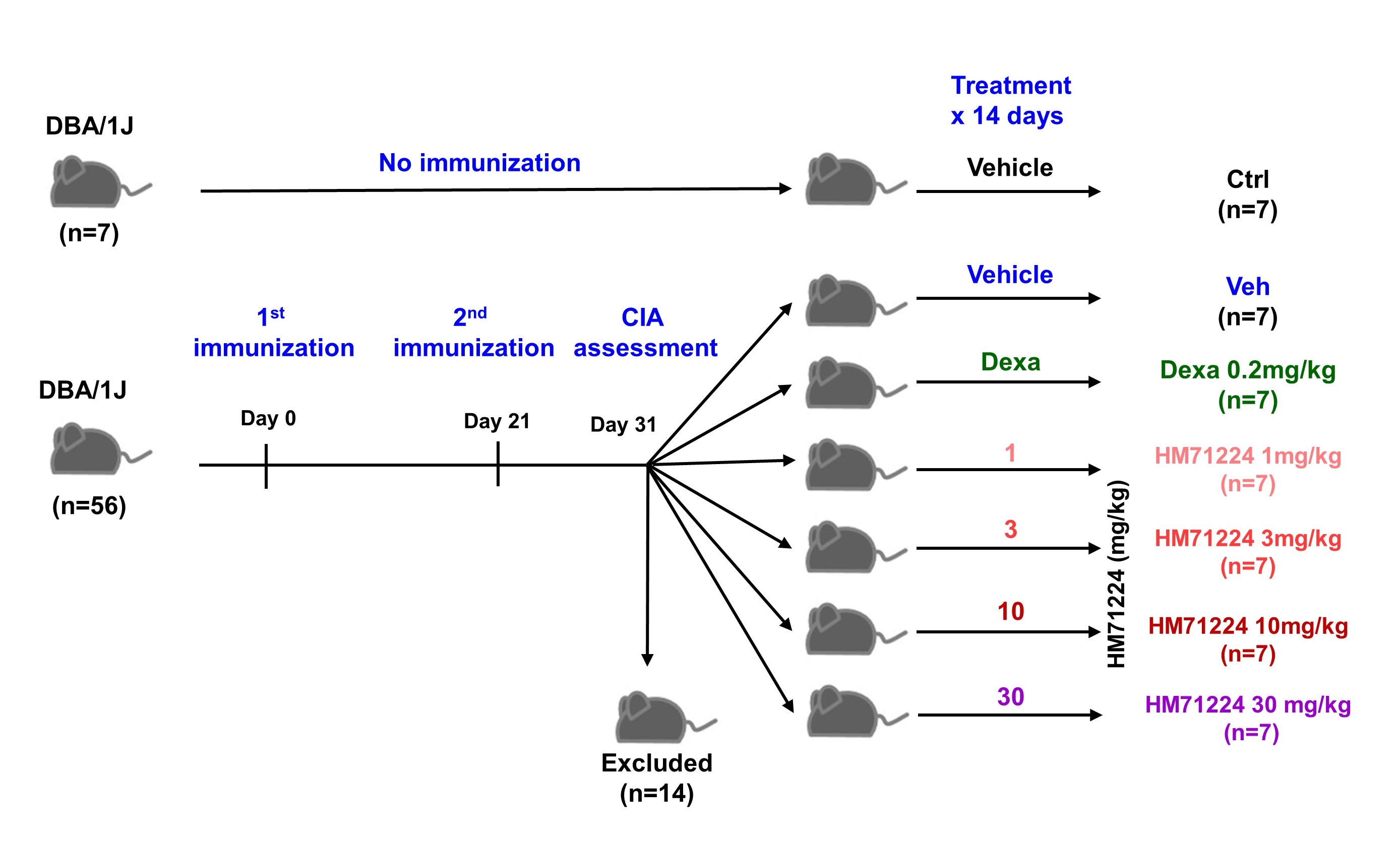
**

**Figure S2. Effect of HM71224 on EGFR signaling. A**, HM71224 does not inhibit proliferation of EGFR-overexpressing epidermoid carcinoma A431 cell line (GI_50_ = 1000 nM). **B**, IC_50_ of HM71224 on inhibiting EGFR phosphorylation was 800 nM.

**
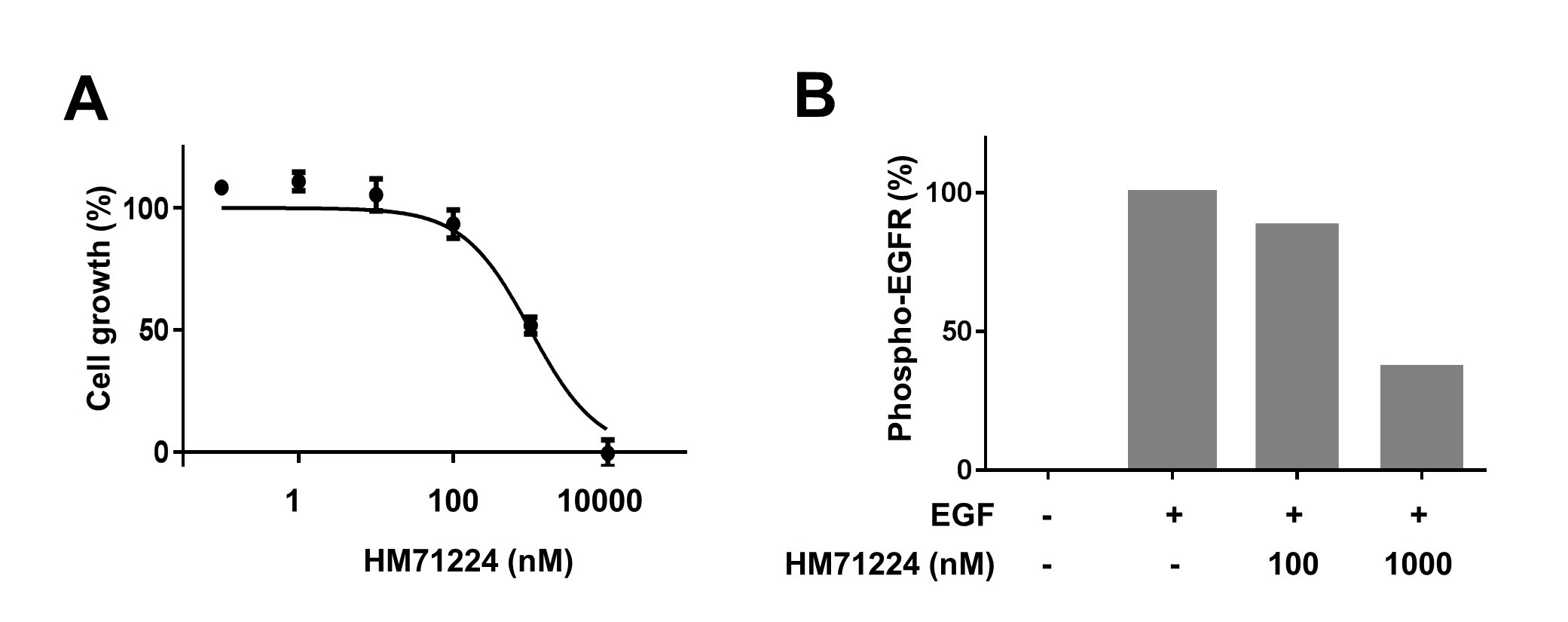
**

**Figure S3. HM71224 inhibits Btk phosphorylation.** Human PBMCs were treated with increasing concentrations of HM71224. Following cross-linking the BCR with anti-IgM F(ab’)_2_, phosphorylation status of Btk, PLC-γ2, Erk was measured by phospho-flow cytometry. HM71224 inhibited phosphorylation of BTK, and p-Erk in a dose-dependent manner.


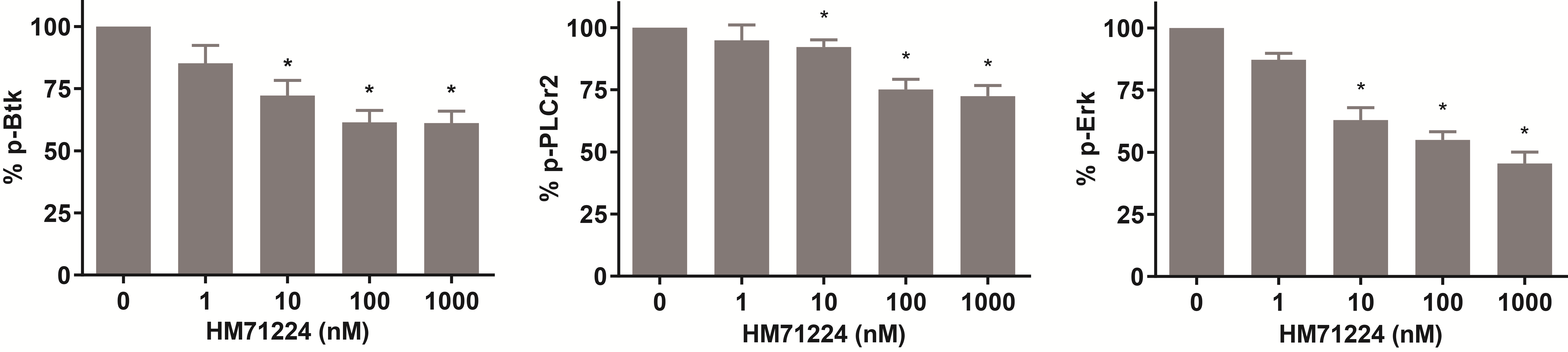


**Figure S4.** HM71224 inhibits osteoclastogenesis. A. *In vitro* osteoclastogenesis assay of mouse bone marrow cultures, followed by TRAP staining. B. Number of osteoclasts in cultures of mouse bone marrow macrophages after exposure to the indicated doses of HM71224.

**
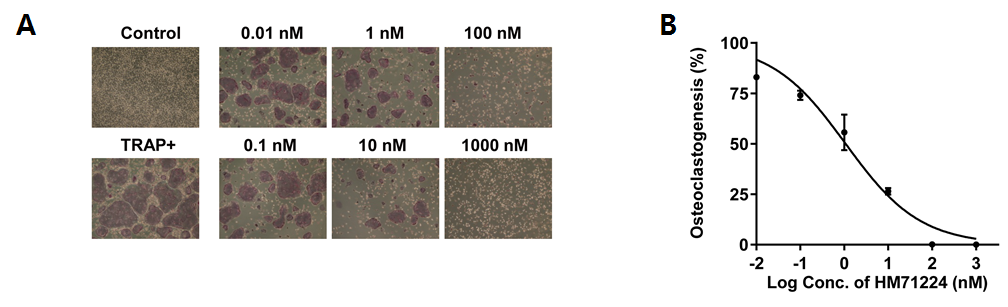
**

**Figure S5. Effect of HM71224 on human T cell signaling.** PBMCs isolated from healthy donors were activated with CD3- and CD28- conjugated beads in the presence of HM71224, PCI-32765 (Ibrutinib), Tofacitinib (a JAK inhibitor) or placebo. The expression level of p-STAT1, p-STAT3, p-STAT5, and p-ERK1/2 by CD3+ T cells was then measured by phospho-flow cytometry. T cell receptor signaling (p-STAT1, p-STAT3 and p-STAT5) was not affected by HM71224 or PCI-32765, while they were significantly suppressed by Tofacitinib. p-ERK1/2 was inhibited by both HM71224 and PCI-32765.


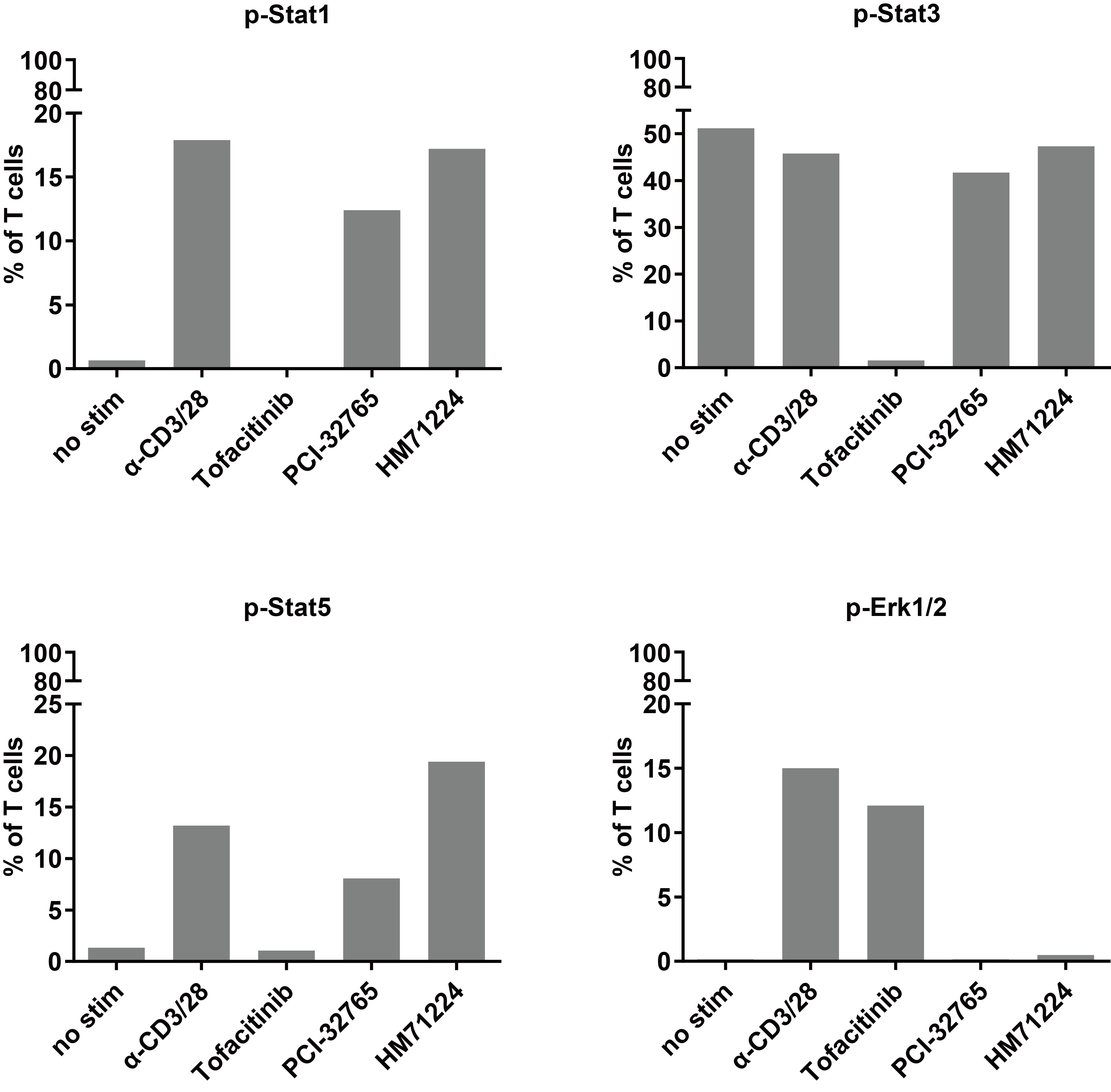

Supplement: Additional file 1: Table S1. — Sequence alignment of kinases. Table S2. Arthritis score and body weight during treatment period. Figure S1. Experimental design of collagen-induced arthritis. Figure S2. Effect of HM71224 on EGFR signaling. Figure S3. HM71224 inhibits Btk phosphorylation. Figure S4. HM71224 inhibits osteoclastogenesis. Figure S5. Effect of HM71224 on human T cell signaling. (DOCX 1545 kb) [file 13075_2016_988_MOESM1_ESM.docx]
